# Non-*Saccharomyces* in Wine: Effect Upon *Oenococcus oeni* and Malolactic Fermentation

**DOI:** 10.3389/fmicb.2018.00534

**Published:** 2018-03-23

**Authors:** Aitor Balmaseda, Albert Bordons, Cristina Reguant, Joaquín Bautista-Gallego

**Affiliations:** ^1^Departament de Bioquímica i Biotecnologia, Facultat d’Enologia, Universitat Rovira i Virgili, Catalonia, Spain; ^2^Food Biotechnology Department, Instituto de la Grasa, Consejo Superior de Investigaciones Científicas, Universidad Pablo de Olavide, Seville, Spain

**Keywords:** non-*Saccharomyces*, malolactic fermentation, *Oenococcus*, wine, microbial interactions

## Abstract

This work is a short review of the interactions between oenological yeasts and lactic acid bacteria (LAB), especially *Oenococcus oeni*, the main species carrying out the malolactic fermentation (MLF). The emphasis has been placed on non-*Saccharomyces* effects due to their recent increased interest in winemaking. Those interactions are variable, ranging from inhibitory, to neutral and stimulatory and are mediated by some known compounds, which will be discussed. One phenomena responsible of inhibitory interactions is the media exhaustion by yeasts, and particularly a decrease in L-malic acid by some non-*Saccharomyces*. Clearly ethanol is the main inhibitory compound of LAB produced by *S. cerevisiae*, but non-*Saccharomyces* can be used to decrease it. Sulfur dioxide and medium chain fatty acids (MCFAs) produced by yeasts can exhibit inhibitory effect upon LAB or even result lethal. Interestingly mixed fermentations with non-*Saccharomyces* present less MCFA concentration. Among organic acids derived as result of yeast metabolism, succinic acid seems to be the most related with MLF inhibition. Several protein factors produced by *S. cerevisiae* inhibiting *O. oeni* have been described, but they have not been studied in non-*Saccharomyces*. According to the stimulatory effects, the use of non-*Saccharomyces* can increase the concentration of favorable mediators such as citric acid, pyruvic acid, or other compounds derived of yeast autolysis such as peptides, glucans, or mannoproteins. The emergence of non-*Saccharomyces* in winemaking present a new scenario in which MLF has to take place. For this reason, new tools and approaches should be explored to better understand this new winemaking context.

## Introduction

Wine is the result of the alcoholic fermentation (AF) driven out by oenological yeasts in a complex microbial environment (Constantí et al., 1997; Beltran et al., 2002). Apart from *Saccharomyces cerevisiae*, recognized as the main agent of this process, other yeast species, known as non-*Saccharomyces* yeasts, such as *Hanseniaspora/Kloeckera*, *Pichia*, *Candida*, or *Metschnikowia* are implicated in early stages of the AF (Fleet et al., 1984). After the AF, the resultant wine can undergo the malolactic fermentation (MLF), which consists on a fairly simple reaction: a unique enzymatic decarboxylation of the L-malic acid to L-lactic acid (Liu, 2002). It is usually performed in red wines or high acidity white wines. This fermentation is carried out by lactic acid bacteria (LAB). Four LAB genera are usually found in wine: *Lactobacillus*, *Pediococcus*, *Leuconostoc*, and *Oenococcus;* and particularly, the main dominant species in wine is *Oenococcus oeni* (Wibowo et al., 1985; Lonvaud-Funel, 1999; Liu, 2002). MLF is related to a quality improvement in wine since this biotransformation leads to a pH increase, enhanced organoleptic properties and a microbial stabilization (Lonvaud-Funel, 1999). During MLF, LAB consume L-malic acid and other nutrients, impoverishing wine and avoiding the development of contaminant microorganisms.

In the last few years the interest on the use of non-*Saccharomyces* yeasts in winemaking has increased (Padilla et al., 2016; Petruzzi et al., 2017), due to the particular enzymatic activities that catalyze the liberation of aromas from their non-volatile precursors (Belda et al., 2017). Generally, these yeasts are inoculated to start the AF of must and later *S. cerevisiae* is inoculated to finish the process. This type of sequential inoculation with non-*Saccharomyces* undergoes chemical changes in wine which modulate the organoleptic profile of wines (Fleet, 2008; Padilla et al., 2016). What is more, this chemical modulation presents new scenery in which MLF may take place.

The purpose of this mini review is to summarize the current knowledge about the compounds responsible for the interactions that may take place between oenological yeasts and LAB during winemaking, highlighting the new scenery of non-*Saccharomyces* fermentations.

## Yeast-Lab Interactions: Oenological Context

**FIGURE 1:**
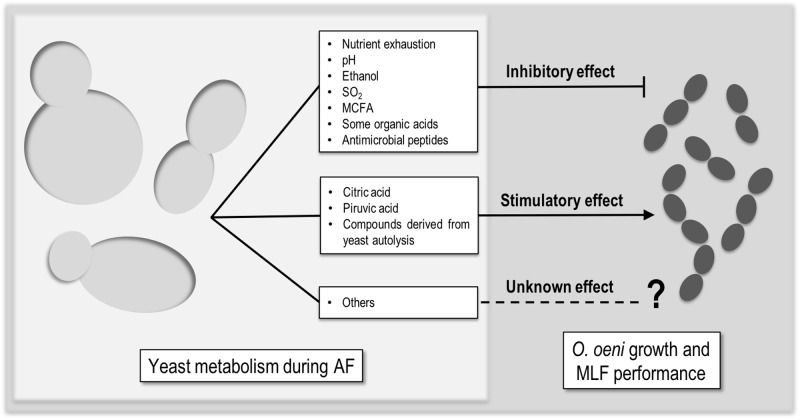
Compounds produced by yeast that can mediate inhibitory, stimulatory, or unknown effect in *Oenococcus oeni* growth or MLF performance.

The performance of MLF by LAB is highly affected by the physicochemical intrinsic properties of wine, such as pH, ethanol, and SO_2_ (Carreté et al., 2002; Arnink and Henick-Kling, 2005). Moreover, since MLF takes place usually after the AF, it is also influenced by yeast metabolism. Those interactions range from inhibitory, to neutral and stimulatory. There is not much literature about this topic, but it is agreed that the type and impact of the interactions is dependent on several factors like (I) the initial must composition, (II) the yeast/bacteria strain combination, (III) the uptake and release of nutrients by yeasts, and (IV) the ability of yeasts to produce metabolites that affect somehow LAB (King and Beelman, 1986; Lonvaud-Funel et al., 1988; Alexandre et al., 2004; Du Plessis et al., 2017). There are some compounds which mediate these interactions (**Figure 1**) but, still the available information is not sufficient.

Up to date, some strategies have been developed to mitigate the possible yeast- *O. oeni* inhibitory interactions (Sumby et al., 2014). Specifically, coinoculation of yeast and *O. oeni* has been proposed as a promising strategy to reduce the length of MLF (Izquierdo Cañas et al., 2014). In this way, the simultaneous AF and MLF co-immobilized in alginate beads is a technique currently in study (Bleve et al., 2016). Another classical approach to deal with the MLF difficulties is to select specific strains from the nature (Campbell-Sills et al., 2017; Petruzzi et al., 2017). The purpose of this selection is to identify the most relevant microorganisms related with the fermentation process in a particular area and use them as culture starters (Portillo et al., 2016; Franquès et al., 2017; Petruzzi et al., 2017).

Above the direct yeasts effect upon LAB and MLF performance, the must, and the winemaking practices, have a strong impact in how these interactions take place (Arnink and Henick-Kling, 2005; Tristezza et al., 2016).

Beyond the particular production of certain compounds (**Table 1**), yeast metabolism exhausts the nutrients of the medium. LAB have complex nutrient requirements (Garvie, 1967; Fourcassie et al., 1992; Terrade and Mira de Orduña, 2009), so their growth is highly dependent on the nutrients consumption during AF by yeasts (Ivey et al., 2013). The effect of these inhibitory interactions could be explained as the result of nutrient competition, such as yeast assimilable nitrogen (YAN) or amino acids (Costello et al., 2003). Therefore, yeast strains with complex nutrient requirements would exhibit an increased antagonistic relationship with LAB (Costello et al., 2003). In this way, it has been recently described that coinoculation of *S. cerevisiae* with other non-*Saccharomyces* yeasts result in a metabolic stimulation of glucose and nitrogen uptake by yeasts, which could lead to a more impoverished medium for LAB (Curiel et al., 2017).

**Table 1:** Main compounds affected (variation in content, negative or positive) by the use of non-*Saccharomyces* in alcoholic fermentation regarding to *S. cerevisiae* as sole starter.

Compound	Non-*Saccharomyces*^∗^	Variation respect to *S. cerevisiae*	Reference
L-Malic acid	*T. delbrueckii*+ *S. c.*	-	Belda et al., 2015
	*S. bacillaris*+ *S. c.*	-	Tofalo et al., 2012; Du Plessis et al., 2017
	*M. pulcherrima*+ *S. c.*	-	Du Plessis et al., 2017
	*I. orientalis*+ *S. c.*	-	Kim et al., 2008
	*Sc. pombe*+ *S. c.*	-	Benito et al., 2013, 2014
Ethanol	*M. pulcherrima*+ *S. c.*	-	Contreras et al., 2014
	*T. delbrueckii*+ *S. c.*	-	Belda et al., 2015
	*C. stellata*+ *S. c.*	-	Ferraro et al., 2000
	*S. bacillaris*+ *S. c.*	-	Masneuf-Pomarede et al., 2015
Sulfur dioxide	*T. delbrueckii*	+	Belda et al., 2015
Medium chain fatty acids	*H. uvarum*+ *S. c.*	-	Liu P.-T. et al., 2016
	*I. orientalis*+ *S. c.*	-	Liu P.-T. et al., 2016
	*T. delbrueckii*+ *S. c.*	-	Belda et al., 2015
	*L. thermotolerans*	-	Shekhawat et al., 2017
	*M. pulcherrima*+ *S. c.*	+	González-Royo et al., 2015; Liu P.-T. et al., 2016
	*C. stella*+ *S. c.*	+	Liu P.-T. et al., 2016
	*P. fermentans*+ *S. c.*	+	Liu P.-T. et al., 2016
Citric acid	*S. bacillaris*+ *S. c.*	+	Giaramida et al., 2013
Pyruvic acid	*T. delbrueckii*	+	Belda et al., 2015
	*T. delbrueckii*+ *S. c.*	+	Belda et al., 2015
	*C. stellata*+ *S. c. L. thermotolerans* + *S. c.*	+	Soden et al., 2000; Jolly et al., 2006; Belda et al., 2015
Glycerol	*T. delbrueckii*+ *S. c.*	+	Benito et al., 2016
	*C. stellata*+ *S. c. L. thermotolerans* + *S. c.*	+	Soden et al., 2000; Jolly et al., 2006; Benito et al., 2016
	*S. bacillaris*+ *S. c.*	+	Englezos et al., 2016b
Mannoproteins	*M. pulcherrima*+ *S. c.*	+	Belda et al., 2016
	*T. delbrueckii*+ *S. c.*	+	González-Royo et al., 2015; Belda et al., 2016

*^∗^S. c. corresponds to Saccharomyces cerevisiae.*

Moreover, it has been reported that the use of some yeast strains (Su et al., 2014) can cause a decrease in L-malic acid, the prior substrate of LAB in wine, which can negatively affect the MLF performance. Particularly, the use of non-*Saccharomyces* leads a higher consumption of L-malic acid, as it has been described with *Torulaspora delbrueckii* (Belda et al., 2015), *Starmerella bacillaris* (syn. *Candida zemplinina*) (Tofalo et al., 2012; Du Plessis et al., 2017), *M. pulcherrima* (Du Plessis et al., 2017), and *Issatchenkia orientalis* (Kim et al., 2008). There is also another non-*Saccharomyces* yeast that really consumes L-malic acid to dryness (Du Plessis et al., 2017). *Schizosaccharomyces* spp. can develop the maloalcoholic fermentation by consuming both sugars and L-malic acid (Benito et al., 2013, 2014).

Alcoholic fermentation of grape must undergoes deep chemical changes enhanced by ethanol and sulfur dioxide. Long ago, it is agreed that concentrations over 4% (v/v) of ethanol inhibit the growth of most LAB (Capucho and San Romao, 1994). Also, a more recent study reported the triad of ethanol, SO_2_ and medium chain fatty acids (MCFAs) as the main inhibitor compounds in the antagonism between yeast and *O. oeni* (Nehme et al., 2008). The main functional categories of genes affected by ethanol are metabolite transport and cell wall and membrane biogenesis (Olguín et al., 2015). Nowadays, some non-*Saccharomyces* yeasts are currently used in mixed fermentations to decrease the alcoholic content of wines (Giaramida et al., 2013; Loira et al., 2014; Ciani et al., 2016), such as *M. pulcherrima* (Contreras et al., 2014), *T. delbrueckii* (Belda et al., 2015), *C. stellata* (Ferraro et al., 2000) and *S. bacillaris* (Englezos et al., 2016a), possibly mitigating the negative effect of ethanol upon LAB growth.

The role of SO_2_ as an antimicrobial compound is known since ancient Romans that used to add this chemical to prevent food and beverage from spoilage. Its active mechanism affects *O. oeni* membrane and causes an ATPase activity decrease (Carreté et al., 2002), causing a delay or the failure of MLF (Lonvaud-Funel et al., 1988). It is customary to use this compound to control microbial communities since vineyard to wine in the winemaking. Moreover, yeasts are able to produce this compound as result of their metabolism (Wells and Osborne, 2011). The common amount of SO_2_ produced by *S. cerevisiae* strains is less than 30 mg/L, but some strains can produce more than 100 mg/L of this compound (Suzzi et al., 1985; Rankine, 1968). When it comes to non-*Saccharomyces* yeasts, there is no much information about their SO_2_ production since they are more affected by this compound (Jolly et al., 2014). However, it has to be pointed that the use of *T. delbrueckii* as sole starter increased the SO_2_ concentration of the final wine (Belda et al., 2015). Apart from the cited strain effect, the medium has great influence in the production of SO_2_ by yeasts. Higher concentration of YAN in must ends on higher amount of SO_2_ (Osborne and Edwards, 2006), as result of the metabolism of the sulfured amino acids.

### Medium Chain Fatty Acids (MCFAs)

During AF, yeasts produce different compounds as result of their growth metabolism that can inhibit *O. oeni* growth and MLF. MCFA (C_8_–C_14_) constitute a group of organic molecules that can limit *O. oeni* growth and even decrease their L-malic consumption (Edwards and Beelman, 1987; Lonvaud-Funel et al., 1988). It has to be mentioned the strong effect of winemaking practices in fatty acids metabolism by yeasts (Guilloux-Benatier et al., 1998). These authors related a fine MLF performance with a large pre fermentative maceration, possibly due to the high macromolecules concentration and long chain fatty acid extraction (Guilloux-Benatier et al., 1995, 1998). The effect of using non-*Saccharomyces* yeasts in the production of MCFA is variable. Strains belonging to *M. pulcherrima*, *C. stella*, and *Pichia fermentans* increase the final concentration of MCFA (Liu P.-T. et al., 2016). In contrast, mixed fermentations with *H. uvarum*, *I. orientalis* present the opposite behavior (Liu P.-T. et al., 2016). Also, a significant decrease in MCFA concentration has been reported by *Lanchacea thermotolerans* as sole starter (Shekhawat et al., 2017). Hu et al. (2018) reported a strong influence in MCFA concentration related with the inoculation timing of *H. uvarum* in mixed fermentation with *S. cerevisiae.* In this experiment inoculation timing seem to determine the increase or decrease in MCFA concentration regarding to *S. cerevisiae* traditional fermentation. Generally, C_12_ and C_14_, as free fatty acids, are the most toxic MCFA for *O. oeni* (Guilloux-Benatier et al., 1998). Moreover, the esterified forms are even more toxic than free fatty acids, being the most toxic esterified MCFA C_10_, C_12_, and C_14_ (Guilloux-Benatier et al., 1998). So, depending on the particular MCFA and its concentration, the inhibitory effect can become lethal to LAB (Edwards and Beelman, 1987).

### Organic Acids Similar to L-Malic Acid

Malolactic fermentation is the consequence of a unique enzymatic activity performed by the malolactic enzyme. Accordingly, structurally similar organic acids will act as competitive inhibitors for the active site of the malolactic enzyme (Lonvaud-Funel and Strasser de Saad, 1982) and probably they will delay the MLF duration. Early studies in this subject related this effect with succinic acid, fumaric acid, citric acid, and tartaric acid (Lonvaud-Funel and Strasser de Saad, 1982; Davis et al., 1985). Among these acids, succinic acid is the most studied since oenological yeasts can largely produce this compound. First studies related the inhibition of MLF by criotolerant *S. cerevisiae* strains which are characterized by high production of succinic acid and β-phenylethanol (Caridi and Corte, 1997). More recent studies agreed with the inhibition effect of succinic acid (Son et al., 2009), and not with its role as MLF extender.

### Citric Acid

Even though citric acid is considered as inhibitor of the malolactic enzyme (Lonvaud-Funel and Strasser de Saad, 1982), citric acid can be catabolized by LAB (Liu, 2002). This metabolic activity is found in some *O. oeni* strains as response to acidity or ethanol stress (Olguín et al., 2009). Due to the consumption of citric acid, diacetyl is produced (Swiegers et al., 2005). It is usually desirable to have strains which can consume citric acid due to the organoleptic complexity that is achieved (Lonvaud-Funel, 1999). In this way, a high concentration of diacetyl is reported as undesirable (Davis et al., 1985; Bartowsky and Henschke, 2004). Moreover, due to the citric acid metabolism, *O. oeni* increases the volatile acidity (Lonvaud-Funel, 1999; Liu, 2002). Even thought, citric acid increases the transmembrane gradient which generate energy in terms of proton-motive force for *O. oeni* (Liu Y. et al., 2016).

Anyway, since citric acid concentration is usually not very high, acetic acid does not increase very much. Citric acid production by yeast is highly species and strain dependent (Fleet, 2008). On the top of that, mixed fermentations with different non-*Saccharomyces* species exhibit particular citric acid production (Jussier et al., 2006; Giaramida et al., 2013; Izquierdo Cañas et al., 2014). For the moment the only mixed fermentation that clearly increased citric acid concentration is with *S. bacillaris* (Giaramida et al., 2013).

### Pyruvic Acid

Pyruvic acid is an intermediary produced by yeast during the AF. This compound can improve MLF performance by *O. oeni*. It acts as external electron acceptor, facilitating the regeneration of NAD^+^ (Maicas et al., 2002). It can also promote diacetyl production (Mink et al., 2015). Related to increasing the concentration of this compound, Belda et al. (2015) reported higher production of pyruvic acid when *T. delbrueckii* was used as sole or mixed culture starter with *S. cerevisiae*. Benito et al. (2016) reported similar results using *L. thermotolerans*.

### Glycerol

The production of glycerol is directly related with the activity of yeasts by the glyceropyruvic fermentation pathway (Ciani and Maccarelli, 1998). Glycerol can be assimilated and degraded by some spoiling *Lactobacillus* in wine (Liu, 2002). On the contrary, there is no literature that reports this behavior when it comes to *O. oeni*. It is unclear how can affect glycerol to *O. oeni*, since it does not assimilate it, neither degrade it. Usually, non-*Saccharomyces* yeasts exhibit higher metabolic activity of this pathway (Ciani and Maccarelli, 1998; Jolly et al., 2006, 2014). Specifically, *T. delbrueckii* (Belda et al., 2015) and *C. stellata* (Soden et al., 2000; Jolly et al., 2006) have been reported as big glycerol and pyruvic acid producers as result of their high glyceropyruvic fermentation activity. Also, mixed fermentations with *S. bacillaris* and *L. thermotolerans* exhibit higher production of glycerol in regards to a conventional *S. cerevisiae* fermentation (Benito et al., 2016; Englezos et al., 2016b).

### Compounds Derived of Yeast Autolysis

One of the most known positive effects upon MLF performance is its development in presence of yeast lees (Guilloux-Benatier et al., 1995). It has been reported that the inhibitory interactions between yeasts and LAB is counteracted by the presence of yeast lees, and even more, the positive interactions are enlarged (Patynowski et al., 2002). During aging, yeasts undergo an autolytic process that result in the release of different compounds. Nitrogenated compounds, such as amino acids, peptides and proteins, are mainly released as result of yeast autolysis (Guilloux-Benatier et al., 1995; Martínez-Rodriguez et al., 2001). The release of such compounds can help to enrich the previously exhausted medium by yeasts (Costello et al., 2003), stimulating the growth of LAB and MLF performance (Guilloux-Benatier et al., 1995; Diez et al., 2010).

Other molecules like glucans and mannoproteins are also released due to this mentioned process and can stimulate LAB growth (Diez et al., 2010). These authors realized that the presence of mannoproteins only exhibited its positive effect on LAB growth when ethanol was present. *O. oeni* can catabolize these mannoproteins and release mannose, which can be substrate of the phosphotransferase system that helps the adaptation of *O. oeni* to the medium (Jamal et al., 2013). Besides this, the impact of the mannoproteins upon LAB was yeast-LAB strain dependent. Recently, it has been reported that some non-*Saccharomyces* strains belonging to *M. pulcherrima* and *T. delbrueckii* release more mannoproteins than *S. cerevisiae* (Belda et al., 2016). Moreover, these molecules could help hijack MCFA present in wine, stimulating LAB growth (Guilloux-Benatier et al., 1995). It has been also been reported that during AF those cited macromolecules are released, depending in the initial colloidal concentration (Guilloux-Benatier et al., 1995). Still, the same study states that the amount of macromolecules released during yeast growth is insignificant in regards to yeast autolysis.

Apart from the mentioned compounds, there are more released compounds during yeast autolysis, such as vitamins, nucleotides and long chain fatty acids, which could be also stimulatory to LAB (Alexandre et al., 2004). Unfortunately, there is no literature currently available about the possible effect of these compounds.

### Other Compounds

In regards to the possible incompatibility between oenological yeasts and LAB, apart from metabolite compounds, the production of antimicrobial proteinaceous compounds by some *S. cerevisiae* strains has been reported. Dick et al. (1992) firstly studied these compounds. They discovered two cationic proteins which were effective against LAB. More recently, another inhibitory protein fraction produced by *S. cerevisiae* CCMI 885 and active against LAB was identified (Branco et al., 2014). In this work, an exhaustive characterization was performed, which resulted in the identification of glyceraldehyde 3-phosphate dehydrogenase (GAPDH) protein fragments. This newly identified antimicrobial peptides with 2–10 kDa size agreed with previously reported antimicrobial peptides (Comitini et al., 2005; Osborne and Edwards, 2007).

There are no studies about these compounds produced by non-*Saccharomyces* yeasts, but some species could present such antimicrobial compounds, like *M. pulcherrima* that produce pulcherrimic acid (Oro et al., 2014), active against other yeasts.

## Future Perspectives

The increasing number of non-*Saccharomyces* species described as beneficial in winemaking demands further investigation of their metabolism. Many factors can influence the effect of non-*Saccharomyces* on wine composition. Besides the yeast species and strain characteristics, the time and the ratio of inoculation, with respect to *S. cerevisiae*, may alter notably the global effect on wine of the use of non-*Saccharomyces*. All these variables may also affect the development of *O. oeni* and MLF. Future research should contribute to a better knowledge of metabolic traits of a wider number of non-*Saccharomyces* strains and their influence on *O. oeni* performance. Among other possible approaches, metabolomics may be a powerful tool to elucidate how the new winemaking scenario of combined yeasts may change MLF evolution.

## Author Contributions

All authors conceived, drafted the manuscript, and approved the final version of the paper.

## Conflict of Interest Statement

The authors declare that the research was conducted in the absence of any commercial or financial relationships that could be construed as a potential conflict of interest.
